# Targeting Lineage-Specific Functions of NR4A1 for Cancer Immunotherapy

**DOI:** 10.3390/ijms26115266

**Published:** 2025-05-30

**Authors:** Jeremy Kleberg, Akhila Nataraj, Yufeng Xiao, Bristy R. Podder, Zeng Jin, Tanzia Islam Tithi, Guangrong Zheng, Keiran S. M. Smalley, Emily K. Moser, Stephen Safe, Chandra K. Maharjan, Ryan Kolb, Weizhou Zhang

**Affiliations:** 1 Department of Pathology, Immunology and Laboratory Medicine, College of Medicine, University of Florida, Gainesville, FL 32610, USA; jkleberg@ufl.edu (J.K.); akhila.nataraj@ufl.edu (A.N.); bristypodder@ufl.edu (B.R.P.); zengjin@ufl.edu (Z.J.); ttithi@ufl.edu (T.I.T.); ryankolb@ufl.edu (R.K.); 2 Department of Biochemistry and Molecular Biology, College of Liberal Arts and Sciences, University of Florida, Gainesville, FL 32610, USA; 3 Department of Health Science, College of Public Health and Health Professions, University of Florida, Gainesville, FL 32610, USA; 4 Department of Medicinal Chemistry, College of Pharmacy, University of Florida, Gainesville, FL 32610, USA; yufengxiao@cop.ufl.edu (Y.X.); zhengg@cop.ufl.edu (G.Z.); 5 UF Health Cancer Center, University of Florida, Gainesville, FL 32610, USA; 6 Department of Tumor Microenvironment and Metastasis, Moffitt Cancer Center, Tampa, FL 33612, USA; keiran.smalley@moffitt.org; 7 Department of Pulmonary, Critical Care and Sleep Medicine, College of Medicine, University of Florida, Gainesville, FL 32610, USA; emily.moser@medicine.ufl.edu; 8 Molecular & Cellular Oncology Laboratory, Physiology and Pharmacology and of Biochemistry and Biophysics, Texas A&M University Veterinary Medicine & Biomedical Sciences, College Station, TX 77843, USA; ssafe@cvm.tamu.edu

**Keywords:** NR4A1, cancer, immunotherapies, immune cells, tumor microenvironment

## Abstract

Orphan nuclear receptor 4A1 (NR4A1, Nur77) plays a crucial role in regulating immune cell metabolism and function within the tumor microenvironment (TME), thus influencing cancer progression and serving as a potential therapeutic target for cancer immunotherapy. A comprehensive review discussing the multifaceted roles of NR4A1 in immune cells and the exploitation of that knowledge for therapeutic development is lacking in the field. This review explores diverse functions of NR4A1 in tumor-associated immune cells, including T cells, monocytes, natural killer cells, B cells, dendritic cells, macrophages, and neutrophils. NR4A1 contributes to immune regulation by impacting cytokine production, cell differentiation, and immune cell exhaustion. We highlight how NR4A1 in immune cells within the TME may be either a positive (e.g., macrophages in colon cancer) or negative prognostic factor (e.g., T cells in melanoma), depending on the cancer and immune cell context. Additionally, this review also highlights potential therapeutic strategies targeting NR4A1, leading to its inhibition, activation, or degradation to restore immune cell function and enhance anti-tumor immunity. Such therapies could potentially improve patient outcomes by altering immune cell behaviors, blocking intrinsic tumor growth pathways, or via both mechanisms. However, the development of NR4A1-targeted therapies will be dependent on further research to better understand lineage-specific roles of NR4A1 and the underlying mechanisms across different cancer types and immune cells.

## 1. Introduction

The tumor microenvironment (TME) is composed of various host cells surrounding the tumor cells [1]. Each constituent cell type plays a critical role in the TME; for example, endothelial cells contribute to the formation of blood vessels, fibroblasts provide growth factors, cytokines, and other extracellular components, and adipocytes release stored energy as free fatty acids that can be taken up by cancer cells [1,2]. Immune cells within the TME are especially significant as their roles may differ, either contributing to the progression and metastasis of cancer or suppressing tumor growth through either their pro- or anti-tumor effects [1].

The TME consists of both innate and adaptive immune cells that affect the rate of tumor metastasis and progression [3]. Innate immune cells within the TME include but are not limited to macrophages, neutrophils, natural killer (NK) cells, dendritic cells, and myeloid-derived suppressor cells [3,4]. These cells primarily work by secreting cytokines that activate or inhibit proximal cells in response to tumor antigens [3]. Adaptive immune cells include T cells—which are primarily activated by dendritic cells, and B cells play key roles in immune regulations within the TME [5,6,7]. Among the many factors known to regulate anti-tumor immune responses, NR4A1 is an emerging critical regulator of many cell types within the TME.

NR4A1 is a member of a sub-family of orphan nuclear receptors, specifically nuclear subfamily 4 or NR4A [8]. This includes NR4A1 (Nur77), NR4A2 (Nurr1), and NR4A3 (Nor-1). These genes play an important role in maintaining cellular homeostasis and pathophysiology [8]. Specifically, NR4A1 is an active player in metabolic processes related to cancer progression, including mediating processes such as glycolysis, fatty acid synthesis, and amino acid metabolism [8,9]. NR4A1 regulates the transcription of its target genes by directly interacting with their promoter sequences [8]. NR4A1 target genes include those involved in metabolic reprogramming, making it a prospective therapeutic target for altering metabolic pathways in cancer [8]. Currently, there is no comprehensive review describing the role of NR4A1 in immune cells within the TME, nor is there a comprehensive discussion of the potential context-dependent mechanism of action for the therapeutic targeting of NR4A1 with different drug moieties. Hence, we review the literature on the expression of NR4A1 in different immune cells within the TME and examine how different immune-modulatory genes regulated by NR4A1 alter the activity of immune cells in both a pro- and anti-tumor fashion (Table 1).

## 2. Cell Types

### 2.1. T Cells

#### 2.1.1. T Cell Function in the TME and Its Regulation

T cells, including differentiated CD4^+^ helper T cells and CD8^+^ cytotoxic T cells, elicit an anti-tumor effect in the TME by directly killing cancer cells in an antigen-specific manner and combating tumor growth through the release of cytokines [27]. However, T cell function in the TME can be impaired via several tumor-intrinsic functions. Some tumors are weakly immunogenic with the limited infiltration of T cells [28]. Furthermore, T cells can become less efficient due to exhaustion and dysfunction within the TME [27,29]. One way this phenomenon can occur is via higher expression of programmed death-1 (PD-1) on T cells as a result of constant exposure to antigens and harsh conditions within the TME [27]. PD-1 expression leads to T cell exhaustion and dysfunction and is associated with tumor growth and metastasis [27]. Programmed death-ligand 1 (PD-L1) is a surface protein expressed by tumor cells, myeloid cells, and other stromal cells within the TME [30]. PD-L1 interacts with PD-1 on CD8^+^ T cells, resulting in T cell exhaustion [30]. Therefore, PD-L1 expression limits T cell activation, leading to immune suppression and enhanced tumor progression [31,32]. PD-L1 expression in tumor cells is positively regulated by interferon gamma (IFN-γ) secreted by activated T cells and other antigen-presenting cells [31]. A specific subset of immunosuppressive T cells, called regulatory T cells (Tregs), plays a role in promoting peripheral tolerance by maintaining homeostatic levels of T cell activation within the body [33]. Within the TME, Tregs can suppress the functions of other T cells by secreting tumor growth factor-beta (TGF-β), interleukin-10 (IL-10), and IL-35, which suppress antigen presentation in the tumor and decrease anti-tumor immune responses [33,34].

T cell proliferation and differentiation within the TME and tumor-draining lymph nodes (TDLNs) are dependent on IL-2 [35]. Upon T cell receptor activation, transcription factors such as activator protein 1 (AP-1), nuclear factor kappa-B (NF-κB), and nuclear factor of activated T cells (NFATs) are upregulated, leading to increased IL-2 production [35,36]. In both CD4^+^ helper T cells and CD8^+^ cytotoxic T cells, IL-2 signaling drives differentiation, clonal expansion, and cytokine production, contributing to immune responses [36]. IL-2 is also essential for the development, maintenance, and functions of Tregs [37]. Since Tregs do not produce IL-2, they rely on exogenous IL-2 produced by CD4^+^ T cells and express high levels of the IL-2 receptor alpha (IL-2Rα) subunit, which forms the high-affinity IL-2R complex along with IL-2Rβ and IL-2Rγ [37,38,39].

#### 2.1.2. Role of NR4A1 in T Cell Biology

**Figure 1:**
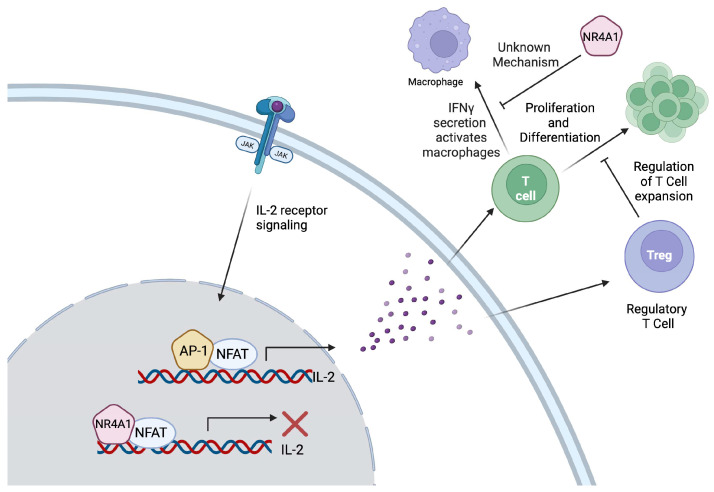
Due to its structural similarity to activator protein-1 (AP-1), NR4A1 competes with AP-1 for binding to AP-1 cis elements, leading to the inhibition of interleukin-2 (IL-2) expression. The production of IL-2 activates both Tregs and T cells. T cell proliferation and differentiation is regulated by regulatory T cells (Tregs); thus, the tight regulation of IL-2 maintains the homeostatic balance. T cells can secrete interferon gamma (IFN-γ) to activate antigen presentation by macrophages, a process that is inhibited by NR4A1, affecting the immune response. However, the mechanistic link between NR4A1 and IFN-γ has not been discovered.

NR4A1 plays multiple roles in different T cells populations within the TME that affect tumor immune responses. The overexpression of NR4A1 in T cells inhibits their differentiation within the TME [20]. It has been shown that overexpression of NR4A1 suppresses effector T cell function, whereas NR4A1 deletion disrupts T cell tolerance, amplifies effector function, and enhances immune responses against tumors and chronic viral infections [20]. NR4A1 and AP-1 have a similar DNA binding domain sequence; thus when overexpressed in T cells, NR4A1 can compete with AP-1 and block the transcriptional activation of IL-2 [20]. NR4A1 also reduces the cytotoxic functions of CD8^+^ T cells by inhibiting their expression of IFN-γ [20]. IFN-γ plays an important role in macrophage activation, phagocytosis and antigen presentation; thus, the NR4A1-mediated suppression of IFN-γ limits CD8^+^ T cell activation and anti-tumor immune responses [40]. However, the mechanism behind the inhibition of IFN-γ expression by NR4A1 is not clearly understood. Additionally, in solid tumors, the expression of NR4A1 contributes to an exhausted state for chimeric antigen receptor T cells through the continued production of T cell exhaustion genes, including PD-1 and the decreased production of effector cytokines [21]. In concordance with this, the deletion of NR4A1 in T cells results in increased T cell effector function and enhanced tumor suppression through the increased production of IL-2 and IFN-γ and decreased PD-1 expression (Figure 1) [20]. Furthermore, in melanoma, colon, and lung cancers, inhibiting NR4A1 in cytotoxic CD8^+^ T cells results in a decrease in tumor infiltration and progression [21,22]. Additionally, the depletion of NR4A1 and NR4A2 in CD8^+^ T cells reduces exhaustion by increasing TCF1 expression in the precursor cells. This enhances their stem-like properties, promoting long-term persistence and expansion within the TME [41]. Beyond CD8^+^ T cells, in colon cancer and lung carcinoma models, NR4A1 inhibition has been shown to decrease the suppressive function of Tregs through the reduced expression of CD25 and cytotoxic T-lymphocyte-associated antigen 4 (CTLA4). In another study, when triple negative breast cancer was treated with an NR4A1 inhibitor, it led to a decrease in the number of tumor-infiltrating Tregs and a decrease in PD-L1 expression [42]. Decreased suppression by Tregs leads to an increased T cell infiltration of the tumor and increased anti-tumor immune responses [43]. Moreover, in a colon cancer model, an NR4A1 antagonist decreased levels of NR4A1, Tox, Tox 2, and NFAT while increasing cytokine production in CD8^+^ T cells, suggesting that the overexpression of NR4A1 drives T cell exhaustion [22]. Thus, NR4A1 in T cells can decrease effector functions and increase immune suppression via multiple mechanisms (Figure 1).

### 2.2. Monocytes

#### 2.2.1. Monocyte Types and Functions in TME

Monocytes play a diverse and sometimes contrasting roles within the TME. They can promote tumor growth through the induction of immune tolerance and supporting angiogenesis, or they can exert anti-tumor effects via their capability to promote immune infiltration and immunosurveillance [11,43]. Circulating monocytes can be separated into three subsets: classical “inflammatory” C-C chemokine receptor type 2 (CCR2)^high^CD14^high^CD16^−^ (CCR2^high^Ly6C^+^ in mice) monocytes, nonclassical “patrolling” C-X3-C motif chemokine receptor 1 (CX3CR1)^high^CD14^low^CD16^+^ (CX3CR1^high^Ly6C^−^ in mice) monocytes, and intermediate CCR2^high^CD14^high^CD16^int^ (Ly6C^int^ in mice) monocytes [44,45,46]. Classical monocytes are involved in inflammatory responses and are recruited to sites of inflammation, such as developing tumors, where they differentiate into macrophages and promote inflammation [43]. These monocytes can promote tumor growth and metastasis [47,48,49]. Patrolling monocytes, in contrast to classical inflammatory monocytes, aid in the resolution of inflammatory responses via “patrolling” vasculature to remove microparticles and cellular debris in a CX3CR1-dependent manner [50,51,52,53]. Patrolling monocytes have been shown to play cancer immunosurveillance roles in the microvasculature of the lungs by recruiting NK cells [54]. Intermediate monocytes have the ability to differentiate to either classical or non-classical monocytes. The role of intermediate monocytes in TME is not completely understood; however, there are studies showing both immunosuppressive (thus pro-tumor) and anti-tumor activities in different contexts [18,43].

#### 2.2.2. Role of NR4A1 in Monocytes

**Figure 2:**
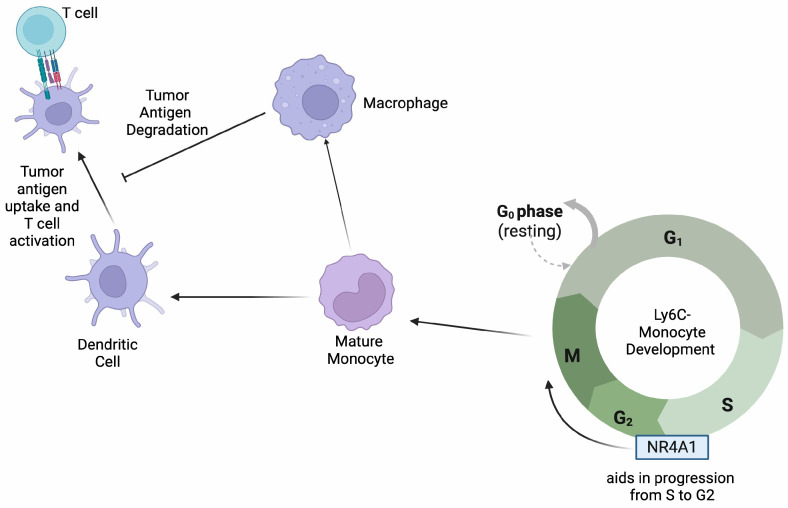
NR4A1 aids in the progression of monocytes through the S/G2 phase of their cell cycle. Mature monocytes differentiate into macrophages and dendritic cells that play specific roles in antigen presentation to T cells as depicted.

NR4A1 has previously been shown to be highly expressed in patrolling monocytes and is essential for their survival [10]. Indeed, mice lacking NR4A1 do not have patrolling monocytes [10]. The depletion of NR4A1 led to the arrest of patrolling monocytes within the S and G2 cell cycle phases; therefore, they were unable to proliferate and differentiate (Figure 2) [10]. Furthermore, the depletion of NR4A1 was correlated with a lower expression of CX3CR1, CCAAT enhancer binding protein beta (C/EBP-β), and JunB, three essential proteins for patrolling monocyte differentiation, resulting in the dysregulation of their development and differentiation [10]. Additionally, the NF-κB signaling pathway was upregulated in NR4A1-depleted mice in patrolling monocytes, leading to the potentiation of their inflammatory phenotype [10]. Hanna RN et al. found that the loss of patrolling monocytes in NR4A1-deficient mice led to increased lung metastasis [11]. The transfer of NR4A1-positive patrolling monocytes into these mice resulted in reduced metastasis via the removal of tumor cells from the lung vasculature and the recruitment of natural killer cells [11]. A follow-up study from the same lab showed that NR4A1 expression in patrolling monocytes was dependent on an enhancer within the NR4A1 promoter that binds to Krüppel-like factors (KLFs) transcription factors [12]. The deletion of this enhancer resulted in the loss of patrolling monocytes and increased metastasis while maintaining NR4A1 expression in macrophages [12]. These data indicate that NR4A1 is required for the differentiation and survival of patrolling monocytes, which may be important for tumor immunosurveillance.

### 2.3. Natural Killer Cells

**Figure 3:**
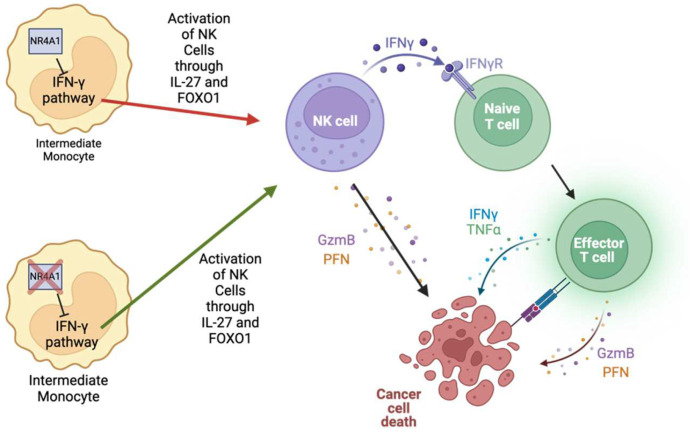
NR4A1 inhibits the IFN-γ pathway within intermediate monocytes, blocking the activation of NK cells via IL-27 and FOXO1. NK cells can directly kill tumor cells by releasing granzyme B and perforin or by activating T cells via IFN-γ secretion. Thus, activated T cells can further augment cancer cell death.

Natural killer (NK) cells within the TME perform multiple roles, including interacting with other immune cells to enhance the immune response against cancer and producing cytotoxic molecules such as granzymes and perforins that directly target and suppress tumor growth and metastasis [55]. Tumor-associated NK cells, a subset of NK cells characterized as dysfunctional due to decreased cytotoxic molecule expression, possess higher NR4A1 expression, and this is similar to the high expression of exhausted CD8^+^ T cells in tumor-infiltrating lymphocytes; however, the underlying mechanism of how NR4A1 leads to this dysfunctional phenotype is not understood [16]. Similar findings were observed in head and neck squamous cell carcinomas, where the overexpression of NR4A1 in NK cells is linked to impaired NK cell activity and increased tumor progression. However, the precise mechanism of how NR4A1 contributes to NK cell dysfunction was not elucidated [17]. In lung cancer models, NR4A1-deficient mice had increased NK cells within the TME and decreased metastasis [10]. Later studies found that NR4A1 in NK cells can inhibit the IFN-γ pathway in intermediate monocytes, prohibiting the monocytes from activating NK cells through IL-27 and FOXO1 [18]. Furthermore, in hepatocellular carcinoma, NR4A1 overexpression in NK cells drives their exhaustion, promoting tumor growth and metastasis by suppressing IFN-γ production and hindering additional immune cell activation against the tumor, such as T cells (Figure 3) [19,40].

### 2.4. B Cells

**Figure 4:**
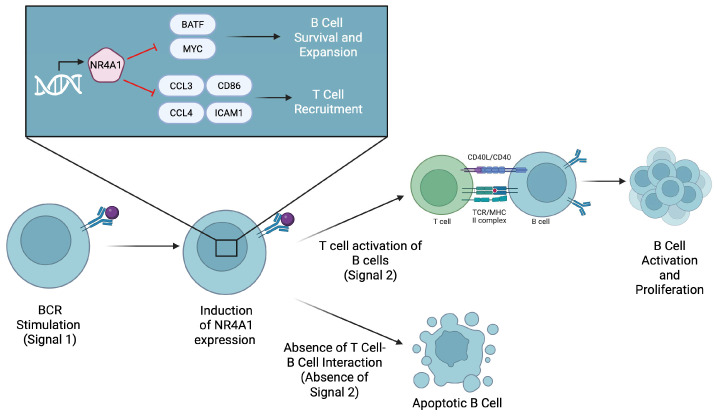
The expression of NR4A1 is induced after B cell receptor (BCR) stimulation by an antigen. NR4A1 then represses BATF and MYC to limit B cell survival and expansion. It also inhibits the expression of CCL3, CCL4, and CD86, which are required for T cell recruitment and co-stimulation. If B cells do not receive timely T cell help, they undergo apoptosis.

The role of B cells within the TME is variable based on the cancer and B cell population types. B cells can be coupled with T cells such that B cells work to promote the recruitment and differentiation of various T cell subsets, which work towards reducing tumor progression [56]. Furthermore, tumor-infiltrating B cells play a role in combating tumor growth and metastasis through the secretion of TRAIL and granzyme B [23]. A limited number of studies have indicated a role for NR4A1 in regulating B cell activity in relation to cancer. In a recent publication from our group, the degradation of NR4A1 using a novel NR4A1 proteolysis targeting chimera (PROTAC) in melanoma models increased tumor-infiltrating B cells, increased CD8^+^ effector memory cells, decreased monocytic myeloid-derived suppressor cells, and reduced tumor growth and metastasis [26]. After treatment with the PROTAC, there was an increase in CD38^+^CD138^−^ plasmablast-like cells and the B cell receptor isotypes were IgD^+^IgM^−^ or IgD^+^IgM^+^ [26]. In contrast, the overexpression of NR4A1 in B cell lymphomas was associated with reduced tumor growth and increased rates of tumor cell apoptosis, while the low expression of NR4A1 led to increased tumor growth and poor clinical outcomes [24]. Mechanistically, NR4A1 promotes the transcription of the apoptotic genes—tumor necrosis factor (TNF)-related apoptosis-inducing ligand (TRAIL), Bim, and p53 upregulated modulator of apoptosis (PUMA) [24]. A study has shown that NR4A1 negatively regulates B cell responses by repressing BATF and MYC as well as cytokines for T cell recruitment (required for the co-stimulatory signals) [25]. The repressed expression of these molecules therefore limits the time window during which B cells can survive and proliferate after antigen stimulation, enforcing a dependence on rapid co-stimulatory signals from CD4^+^ T helper cells [25]. NR4A1 and other NR4A genes limit the expression of C-C motif chemokine ligand 3 (CCL3), CCL4, CD86, and intracellular adhesion molecule 1 (ICAM1), which are crucial for B cell and T cell interactions (Figure 4) [25]. The decreased expression of these molecules makes it difficult for B cells to interact with T cells, causing a decreased immune response against the tumor.

### 2.5. Macrophages and Neutrophils

**Figure 5:**
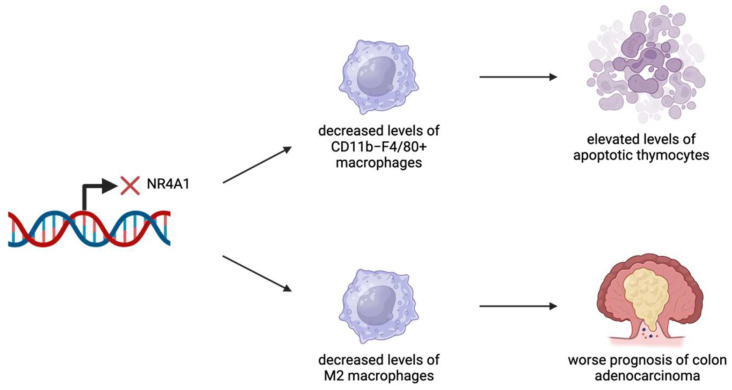
NR4A1 has been identified as an important regulator in several cancer types. NR4A1 expression has been linked to effective levels of macrophages. In the thymus, the lack of NR4A1 expression leads to decreased levels of CD11b^−^F4/80^+^ macrophages, resulting in a greater presence of apoptotic cells. In the colon, the lack of NR4A1 expression leads to decreased levels of M2 macrophages, resulting in worse outcomes related to colon adenocarcinomas.

Macrophages are vital for the regulation and operation of the immune system [57]. Macrophages undergo polarization to different states depending on the cellular context, with M1-like and M2-like macrophages representing the most commonly used nomenclature to describe macrophage polarization [57]. Studies have shown that M1-like and M2-like macrophages have opposing effects within the TME [58]. M1 macrophages are pro-inflammatory in nature and assist in antigen presentation to activate other parts of the immune system [59]. In contrast, M2 macrophages are anti-inflammatory and are poor antigen presenters, causing them to have immunosuppressive effects [58]. A few studies have found a role for NR4A1 in macrophages related to both their physiological and pathological functions in cancer. CD11b^−^F4/80^+^ macrophage cells in the thymus are responsible for degrading apoptotic thymocytes [15]. Mice lacking the expression of NR4A1 transcription factors (*Nr4a1*^−/−^) were unable to develop an adequate level of macrophages to carry out this process, demonstrating the importance of NR4A1 (Figure 5) [15]. Furthermore, NR4A1-deficient mice were shown to experience an “accelerated thymic demise” and an increase in the production of pro-inflammatory cytokines [15]. Combined with an elevated production of anti-nuclear antibodies in *Nr4a1*^−/−^ mice, there is a possibility that macrophages relying on NR4A1 play an important role in thymic homeostasis [15].

NR4A1 has been identified as a potential therapeutic target for colon adenocarcinoma [13]. Patients with elevated levels of tumor-infiltrating M2 macrophages or neutrophils showed poorer prognosis and outcomes [13]. By developing models to predict patient risk based on immune infiltration patterns of these cell types, NR4A1 was found to play a role in regulating tumor survival and relapse [13]. Tumor samples exhibited higher expression levels and a stronger staining intensity of NR4A1 compared to normal colon samples [13]. Overall, elevated NR4A1 expression was linked to better prognosis, partly due to its role in impairing differentiation into M2 macrophages [13]. While neutrophil presence was associated with poorer outcomes, the study did not directly indicate that NR4A1 influences neutrophil differentiation [13].

NR4A1 has otherwise not been specifically linked to any major process or functions in neutrophils [14]. Rather, NR4A2 and NR4A3 have been identified as important regulators of the lifespan of neutrophils and employ mechanisms dependent on protein kinase A and cyclic adenosine monophosphate [14]. More research needs to be conducted to further study the function of NR4A1 in neutrophils.

### 2.6. Dendritic Cells

Dendritic cells (DCs) within the TME play a role in antigen presentation and immune infiltration, helping to initiate immune responses against tumors [60]. By presenting antigens to both CD8^+^ cytotoxic T cells and CD4^+^ helper T cells, DCs contribute to anti-tumor activity [61]. However, tumors can counteract DC function through the secretion of inflammatory molecules/metabolites, such as IL-6 and lactic acid, which inhibit DC differentiation and function [62,63]. Additionally, tumor cells can express CD47, which binds to the signal regulatory protein alpha on DCs, reducing their effectiveness [62,63]. Because of their critical role in shaping immune responses within the TME, DCs are frequently targeted in immunotherapies [9,64]. Understanding the role of transcription factors, such as NR4A1, that regulate DC function is crucial for developing strategies to modulate their activity and enhance therapeutic outcomes [9].

**Figure 6:**
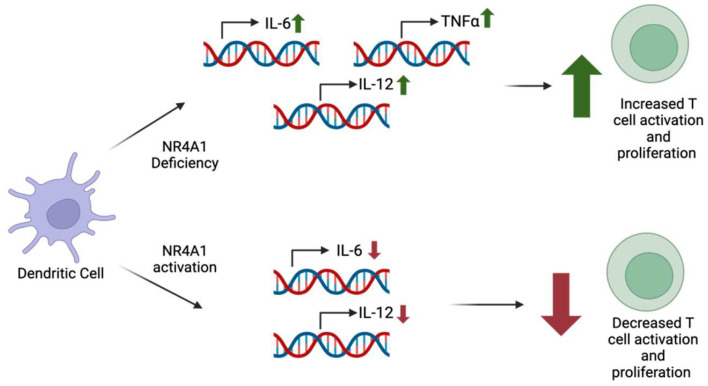
NR4A1 deficiency within dendritic cells has been correlated with increased levels of pro-inflammatory cytokines IL-6, IL-12, and TNF-α. The cytokine levels positively correlate with T cell activation and proliferation. However, NR4A1 in dendritic cells was seen to decrease IL-6 and IL-12 production, leading to decreased T cell activation and proliferation.

Deficiency of NR4A1 in DCs results in the increased production of IL-6, TNFα, and IL-12, whereas the activation of NR4A1 results in reduced IL-6 and IL-12 production [9]. The increased production of these pro-inflammatory cytokines in NR4A1-deficient DCs was associated with heightened T cell activation and proliferation [9]. On the other hand, the activation of NR4A1 leads to decreased levels of IL-6 and IL-12, resulting in the decreased activation and proliferation of T cells (Figure 6) [9]. NR4A1 affects cytokine production by producing changes to the NF-κB signaling pathway [9]. Previous studies have shown that NR4A1 is able to affect this pathway in multiple ways, such as by interacting with the p65 subunit of NF-κB or the regulation of TNF receptor-associated factor 6 (TRAF6) auto-ubiquitination, which plays a major role in signal transduction for NF-κB [9]. Further research is necessary to determine the specific mechanism behind NR4A1′s impact on the NF-κB pathway in DCs and the role of NR4A1 in DCs within a cancer-specific context.

### 2.7. Potential Therapeutic Interventions

Given that NR4A1 promotes the progression of certain cancers, therapies targeting the protein have been investigated and found to augment anti-tumor immunity. As reviewed above, NR4A1 limits the anti-tumor activity of tumor-infiltrating CD8^+^ T cells, NK cells, and B cells, while enhancing the immunosuppressive functions of Tregs [26,65]. Considering such important roles of NR4A1 within the TME and surrounding immune cells, several potential NR4A1 ligands have been developed. These regulators include conventional small molecular inhibitors/activators and heterobifunctional proteolysis targeting chimera (PROTAC) degraders. By using these molecular tools, the therapeutic potential of NR4A1 targeting in cancer therapy and the resultant immunological responses have been explored.

#### 2.7.1. Small-Molecule Inhibitors of NR4A1

Many conventional small molecular ligands including 1,1-bis(3′-indolyl)-1-(p-hydroxyphenyl) methane (DIM-C-pPhOH), kaempferol, quercetin, CCE9, celastrol, etc., have been identified as inhibitors of NR4A1 that can impact both tumor intrinsic and extrinsic cancer immune responses [66,67,68,69]. DIM-C-pPhOH was found to decrease tumor growth by inhibiting the phosphorylation of NR4A1, therefore allowing SMAD7 level to persist within the cell [66]. Studies in rhabdomyosarcoma have shown that kaempferol and quercetin deplete NR4A1 through the inhibition of the mTOR pathway [67]. CCE9 was shown to activate the p38α MAPK pathway in liver and cervical cancers, leading to the mitochondrial localization of NR4A1 [68]. This then induces Bcl-2 phosphorylation and interaction with NR4A1, leading to cell apoptosis [68]. Another inhibitor of NR4A1 is celastrol [68]. Studies have shown that celastrol acts on NR4A1 to increase mitochondrial autophagy and ubiquitination, decreasing an inflammatory response through the NF-κB pathway, specifically in liver cancer models [69]. Celastrol has furthermore been shown to decrease tumor growth and proliferation through its anti-inflammatory and apoptotic inducing effects [70]. Celastrol induces apoptosis through the inhibition of peroxiredoxin 2, an antioxidant enzyme that inhibits apoptosis, leading to increased levels of reactive oxygen species and apoptosis in gastric cancer cells [71]. While many antagonists exist, they lack specificity, and novel therapeutic strategies for targeting NR4A1, such as molecular PROTAC degraders, have been explored.

#### 2.7.2. Direct NR4A1 Degradation via PROTAC Therapy

PROTACs constitute an emerging modality in drug discovery. A PROTAC is a heterobifunctional molecule that comprises a ligand for the target protein, a ligand for an E3 ubiquitin ligase, and a linker that covalently links these two together. Unlike conventional inhibitor-based therapies that inhibit the function of a target protein, PROTACs catalytically degrade a protein of interest (POI) via E3 ubiquitin ligase recruitment, leading to the poly-ubiquitination and degradation of the POI by the ubiquitin–proteasome system (UPS). Therefore, PROTACs offer several advantages over small-molecule inhibitors such as the ability to target traditionally undruggable targets, improved tissue selectivity, and prolonged efficacy [72]. Our previous work has identified NR-V04, an NR4A1-targeting PROTAC, as a potential therapeutic due to its ability to inhibit and degrade NR4A1 [26]. NR-V04 employs celastrol as the NR4A1 ligand and a ligand for VHL E3 ligase [26]. NR-V04 was found to efficiently degrade NR4A1 within the TME and elicit anti-tumor effects consistent with the upregulation of tumor-infiltrating B cells in mouse melanoma models [26]. Despite NR-V04 being a potential new therapeutic agent, further studies need to be conducted in order to determine other effects that NR-V04 may have within the body and to improve its selectivity and druglike properties. Furthermore, considering the cancer-type-dependent TME composition, further research is required to compare the efficacies of NR-V04 and NR4A1 antagonists in other cancer paradigms.

#### 2.7.3. NR4A1 Agonists

While targeting NR4A1 may be beneficial in certain cancers, increasing NR4A1 levels can also prove to be beneficial in other instances [73]. Cytosporone B has been shown to be an agonist of NR4A1 and can lead to the inhibition of tumor growth through the activation of apoptosis [73]. Cytosporone B induces NR4A1 expression, translocation to the mitochondria, and apoptosis through the release of Cytochrome C [73]. NR4A1 has additionally been reported to play a tumor suppressive role in certain acute myeloid leukemia (AML) patients [74,75,76]. Those studies have shown that the acute overexpression or therapeutic induction of NR4A1 in NR4A1-null AML cells can reduce their survival and proliferation [75,76]. Nevertheless, the TCGA-AML dataset shows that either a low or high expression of NR4A1 is equally detrimental to the prognosis and survival of AML patients (ongoing study in our lab). This suggests that NR4A1 could play an oncogenic role in specific AML subsets who could benefit from NR4A1 inhibition or degradation.

NR4A1 is required for the survival and function of patrolling monocytes which help prevent lung metastasis via an immunosurveillance mechanism [11]. Any potential therapy that degrades or inhibits NR4A1 systemically may reduce this population of cells and is something that should be considered in the development of potential NR4A1-targeted therapies. Considering NR4A1 can play a biphasic role depending on the cancer type and TME composition, it will be critical to carefully assess such characteristics to classify patients and predict the therapeutic benefit of NR4A1 modulation.

## 3. Conclusions, Perspectives, and Future Directions

In conclusion, NR4A1 plays a pivotal role in modulating the function of various immune cells within the TME, with context-dependent effects that can either support or hinder tumor progression. The expression of NR4A1 is linked to diverse processes, including the regulation of cytokine production, immune cell differentiation, and immune exhaustion. Its ability to influence the activity of a wide range of immune components makes it a potential target for therapeutic interventions in cancer. While current studies suggest promising outcomes in targeting NR4A1 through approaches such as small-molecule inhibitors or PROTAC-mediated degradation, further research is necessary to fully elucidate its mechanisms across different cancer types and immune cell populations. Understanding the complex interactions of NR4A1 with the immune system could pave the way for novel immunotherapy strategies that enhance anti-tumor immunity and improve clinical outcomes.

One important consideration for NR4A1-targeting therapies is the versatility of the TME components. For example, an NR4A1 PROTAC degrader has been shown to significantly increase tumor-infiltrating B cells, which presumably is responsible for the inhibition of B16F10 melanoma growth in vivo [26]. This is not surprising since B cells are predominant in B16F10 tumors [26]. Other immune cell populations discussed above can also be potentially altered by NR4A1-targeted therapies. However, the net therapeutic response will greatly depend on the dominant immune cell component of the TME and the role of NR4A1 in that compartment. As NR4A1 plays essential roles in immune suppression by maintaining an exhausted state of CD8^+^ T cells/NK cells, the immune suppressive functions of Tregs, the promotion of angiogenesis and cancer progression, it is anticipated that NR4A1-targeted immunotherapy can benefit a broad range of cancer patients in conjunction with current approved immune checkpoint inhibitor-based immunotherapies.

One caveat is the importance of NR4A1 in non-classical, patrolling monocytes that have been known for their functions in killing metastatic cancer cells. Blocking NR4A1 in those cells may promote metastasis. Hence, cancers with increased level of patrolling monocytes should be excluded from NR4A1-targeted cancer immunotherapy.

## Figures and Tables

**Table 1:** Summary of NR4A1 functions in different immune cell types.

Cell Types	Tumor Types	Effects of NR4A1	Mechanism of Action	Summary
Monocytes [10,11,12]	Melanoma and lung	Inhibition of NR4A1 leads to monocytes being trapped in the S/G2 phase of cell cycle, decreasing proliferation and activation.	Decreased NR4A1 is correlated with CX3CR1, JunB, and C/EBP-β, which are essential in monocyte differentiation and proliferation.	Since the depletion of NR4A1 in monocytes leads to decreased proliferation and differentiation, an agonist that can promote its effects within this cell type can aid in combating tumor progression.
Neutrophils [13,14]	Colon adenocarcinomas (COAD)	High NR4A1 expression leads to better prognosis in COAD.	Predictive models identify NR4A1 as a major player in tumor survival.	Depending on NR4A1 expression levels, different therapeutic drugs can be utilized for treatment. Higher levels of NR4A1 expression are associated with better outcomes. More research is needed to determine the impact, if any, of NR4A1 in neutrophil function.
Macrophages [13,15]	Apoptotic thymocytes, COADs	Inadequate levels of macrophages lead to difficulties in degrading apoptotic thymocytes. High NR4A1 expression leads to better prognosis in COAD.	Lack of NR4A1 expression leads to a decrease in macrophage production. Predictive models identify NR4A1 as a major player in tumor survival.	NR4A1 expression is vital for macrophages to successfully degrade apoptotic thymocytes. Depending on NR4A1 expression levels, different therapeutic drugs can be utilized for treatment. Higher levels of NR4A1 expression are associated with better outcomes.
NK Cells [16,17,18,19]	Head and neck squamous carcinomas, lung cancer, hepatocellular carcinoma	Overexpression can lead to tumor progression and increased metastasis.	Overexpression of NR4A1 is associated with decreased production of granzyme and perforin and inhibition of IFN-γ pathway.	Tumor-associated NK cells show an overexpression of NR4A1, which correlates with increased tumor progression and poor prognosis.
Dendritic Cells (DCs) [9]	Acute lymphoblastic leukemia	Activation of NR4A1 leads to decreased inflammatory responses.	Through the regulation of NF-κB pathway, IL-6 and TNFα production is decreased.	NR4A1 expression in DCs can vary depending on immune stimuli, but deficiency has been associated with elevated inflammatory responses. More research is needed to determine specific pathways of mechanisms in different DC subsets.
T cells [20,21,22]	Melanoma, colon, lung, and other solid tumors	Inhibition of T cell development and differentiation.	Blocks IL-2 transcription by competing with AP-1 binding and decreasing IFN-γ production. Contributes to reduced effector cytokine production and increased expression of T cell exhaustion genes.	Since NR4A1 exerts an inhibitory effect on T-cells through the decreased production of IL-2 and IFN-γ, a treatment targeting NR4A1 in these cells can suppress tumor progression.
B cells [23,24,25,26]	B cell lymphoma and melanoma	In B cell lymphoma, overexpression of NR4A1 leads to increased apoptosis and decreased tumor progression. In melanoma studies, degradation of NR4A1 led to increased proliferation of tumor-infiltrating B cells and decreased tumor progression.	In B cell lymphoma, NR4A1 leads to the increased expression of the apoptotic genes TRAIL, Bim, and Puma. In melanoma studies, NR4A1 limits the ability of B cells to be activated without co-stimulatory responses.	In B cell lymphoma, a drug agonist for NR4A1 can lead to increased tumor apoptosis, leading to better clinical results. In melanoma, a drug that inhibits NR4A1 can lead to increased tumor-infiltrating B cells and decreased tumor progression.

## Abbreviations

The following abbreviations are used in this manuscript:

TME	Tumor microenvironment
NR4A1	Nuclear receptor 4A1
COAD	Colon adenocarcinoma
PD-1	Programmed death-1
PD-L1	Programmed death-ligand 1
IFN-γ	Interferon gamma
Tregs	Regulatory T cells
IL-2	Interleukin-2
AP-1	Activator protein-1
NF-κB	Nuclear factor kappa-B
NFAT	Nuclear factor of activated T cell
IL-2Rα	IL-2 receptor alpha
CCR2	C-C chemokine receptor type 2
CX3CR1	C-X3-C motif chemokine receptor 1
C/EBP-β	CCAAT enhancer binding protein beta
KLF	Krüppel-like factors
NK	Natural killer
PROTAC	Proteolysis targeting chimera
TNF	Tumor necrosis factor
TRAIL	Tumor necrosis factor-related apoptosis-inducing ligand
PUMA	p53 upregulated modulator of apoptosis
CCL3	C-C motif chemokine ligand
ICAM1	Intracellular adhesion molecule 1
DCs	Dendritic cells
DIM-C-pPhOH	1,1-bis(3′-indolyl)-1-(p-hydroxyphenyl) methane
POI	Protein of interest
